# Evaluation of the Interrater Reliability of End-of-Life Medical Orders in the Physician Orders for Life-Sustaining Treatment Form

**DOI:** 10.1001/jamanetworkopen.2019.2036

**Published:** 2019-04-12

**Authors:** Gustavo Bigaton Lovadini, Fernanda Bono Fukushima, Joao Francisco Lindenberg Schoueri, Roberto dos Reis, Cecilia Guimarães Ferreira Fonseca, Jahaira Jeanainne Casanova Rodriguez, Cauana Silva Coelho, Adriele Ferreira Neves, Aniela Maria Rodrigues, Marina Almeida Marques, Alessandro Ferrari Jacinto, Karen Harrison Dening, Rick Bassett, Alvin H. Moss, Karl E. Steinberg, Edison Iglesias de Oliveira Vidal

**Affiliations:** 1 Botucatu Medical School, UNESP, Botucatu, Brazil; 2 Dementia UK, London, United Kingdom; 3 Center for Nursing Excellence, St Luke’s Health System, Kansas City, Missouri; 4 Center for Health Ethics and Law, West Virginia University, Morgantown; 5 Institute for Palliative Care, California State University, Long Beach

## Abstract

**Importance:**

Despite its spread in much of the United States and increased international interest, the Physician Orders for Life-Sustaining Treatment (POLST) paradigm still lacks supporting evidence. The interrater reliability of the POLST form to translate patients’ values and preferences into medical orders for care at the end of life remains to be studied.

**Objective:**

To assess the interrater reliability of the medical orders documented in POLST forms.

**Design, Setting, and Participants:**

This cross-sectional study was conducted in a public university hospital in southeastern Brazil. Two independent researchers interviewed the same patients or decision-making surrogates (n = 64) during a single episode of hospitalization within a time frame of 1 to 7 days. Eligible participants were hospitalized adults aged 21 years or older who were expected to remain hospitalized for at least 4 days and whose attending physician responded no to the question, Would I be surprised if this patient died in the next year? Data collection occurred between November 1, 2015, and September 20, 2016, and first data analyses were performed on October 3, 2016.

**Main Outcomes and Measures:**

Interrater reliability as measured by κ statistics.

**Results:**

Of the 64 participants interviewed in the study, 53 (83%) were patients and 11 (17%) were surrogates. Patients’ mean (SD) age was 64 (14) years, and 35 patients (55%) and 8 surrogates (73%) were women. Overall, in 5 cases (8%), disagreement in at least 1 medical order for life-sustaining treatment was found in the POLST form, changing from the first interview to the second interview. The κ statistic for cardiopulmonary resuscitation was 0.92 (95% CI, 0.80-1.00); for level of medical intervention, 0.89 (95% CI, 0.76-1.00); and for artificially administered nutrition, 0.92 (95% CI, 0.83-1.00).

**Conclusions and Relevance:**

The high interrater reliability of the medical orders in POLST forms appears to offer further support for this advance care planning paradigm; in addition, the finding that this interrater reliability was not 100% underscores the need to ensure that patients or their surrogates have decision-making capacity and to confirm that the content of POLST forms accurately reflects patients’ current treatment preferences.

## Key Points

**Question:**

What is the interrater reliability of medical treatment orders documented in the Physician Orders for Life-Sustaining Treatment form?

**Findings:**

In this cross-sectional study of interviews with 64 patients and decision-making surrogates in Brazil, the κ statistics for cardiopulmonary resuscitation, level of medical intervention, and artificially administered nutrition were high. However, disagreement in at least 1 order for life-sustaining treatment was found in 5 cases.

**Meaning:**

The findings support the Physician Orders for Life-Sustaining Treatment paradigm as a means to translate patients’ values and preferences of care at the end of life into medical orders and stress the importance of frequently reviewing the content of the Physician Orders for Life-Sustaining Treatment form to ensure it reflects current preferences.

## Teaser

This cross-sectional study evaluates interrater reliability of medical orders documented in the Physician Orders for Life-Sustaining Treatment (POLST) form to translate the treatment preferences of Brazilian adult patients at the end of life.

## Introduction

The Physician Orders for Life-Sustaining Treatment (POLST) paradigm was created in Oregon in the early 1990s as a coordinated system to elicit, document, and communicate the preferences of patients regarding medical interventions at the end of life.^1,2^ The POLST paradigm was developed with the ethical purpose of increasing the chances of patients’ values and preferences being respected at the end of their lives by the provision of medical care that is consistent with their values. It is primarily intended for patients with limited life expectancy and translates patients’ values and preferences of care into a document (the POLST form), which comprises a standardized set of medical orders concerning life-sustaining interventions. A systematic review of studies about POLST found evidence that preferences of care documented as medical orders in POLST forms are more likely to guide care at the end of life than traditional advance directives alone.^3^ The “Dying in America” report by the Institute of Medicine recognized POLST as an important area of progress toward the provision of end-of-life care that is consistent with patients’ values, and the report recommended the federal government to encourage US states to implement POLST programs.^4^ Within the past decade, POLST has been instituted or is in the process of being implemented in 46 of the 50 states^5^ and has recently raised international interest as a means to promote advance care planning and respect of patients’ values at the end of life.^6^

Despite the recognition of POLST’s importance, there are several gaps in the evidence about POLST.^3,7^ One major underappreciated evidence gap is the absence of studies assessing the interrater reliability of the POLST form to translate patients’ values into medical orders. Assessment of this psychometric property of the POLST form is important because it indicates to what extent one can trust that different clinicians, following a similar advance care planning approach, would arrive at the same set of medical orders documented in a POLST form. Hence, we designed the present study to assess the interrater reliability of the POLST form completion process to capture treatment preferences at the end of life.

## Methods

This cross-sectional study was approved by the ethics research committee of Botucatu Medical School. All participants (ie, patients or their surrogates) signed informed consent forms. Data collection occurred between November 1, 2015, and September 20, 2016, and first data analyses were performed on October 3, 2016.

This study was based on the Consensus-based Standards for the Selection of Health Measurement Instruments^8,9,10^ and followed the Strengthening the Reporting of Observational Studies in Epidemiology (STROBE) reporting guideline^11^ for cross-sectional studies and the Guidelines for Reporting Reliability and Agreement Studies (GRRAS) reporting guideline^12^ for studies of reliability and agreement.

The study was conducted at a single public university hospital in southeastern Brazil. Two of our independent researchers (G.B.L., J.J.C.R., A.M.R., A.F.N., M.A.M., C.G.F.F., C.S.C., J.F.L.S., R.R., and E.I.O.V.) interviewed the same patients or decision-making surrogates during a single episode of hospitalization and within a time frame of 1 to 7 days. The choice of this short time frame was intended to minimize the chance of loss to follow-up and the probability that changes in the state of patient health or care could jeopardize the assessment of interrater reliability.

Patients were eligible for the study if they were aged 21 years or older, were inpatients at the study hospital, and were expected to remain hospitalized for at least 4 days and if 1 of their attending physicians answered no to the following question: Would I be surprised if this patient died in the next year?^13^ If a potentially eligible patient was found during the first interview not to have decision-making capacity but to have a surrogate, the surrogate was invited to participate in the research. Decision-making capacity was determined using the following criteria as described by Appelbaum^14^: (1) ability to communicate a choice, (2) understanding of the information communicated, (3) appreciation for the current medical condition and the likely consequences of different treatment options, and (4) ability to provide a set of reasons for the choice based on personal values. The assessment of decision-making capacity was performed during each advance care planning interview in which interviewers presented patients with a set of clinical situations, treatment options, and possible outcomes and observed how patients dealt with the information given, asked questions, expressed choices, and relayed back their understanding and the reasons for their preferences. Exclusion criteria were (1) the unavailability of the patient to participate in the second interview (eg, because of hospital discharge) and (2) the report in the beginning of the second interview that major clinical or personal events had occurred between interviews that changed the patient’s perspective on end-of-life care.

During the first interview, after patients or their surrogates had signed the consent form, the interviewers collected sociodemographic data on age, sex, race/ethnicity, years of schooling, religion, main diagnosis, Charlson comorbidity index,^15^ and functional status. We used the Palliative Performance Scale (score range: 0%-100%, with 0% indicating death and 100% indicating total autonomy plus lack of active illness) to rate functional status.^16^ After the interview, the interviewers collected clinical data from patients’ medical records, including comorbidities and the principal diagnosis that led to hospital admission.

One third-year medical student (C.S.C.), 3 fourth-year medical students (J.J.C.R., C.G.F.F., and A.F.N.), 3 interns (J.F.L.S., R.R., and A.M.R.), 1 internal medicine resident (M.A.M.), 1 psychiatrist (G.B.M.), and 1 geriatrician (E.I.O.V.) composed the team of interviewers. Interviewers were trained in the structured advance care planning conversation and in the completion of the POLST form. Training sessions were face to face and interactive, took 1 to 1½ hours each, and were provided in small groups or individually. The advance care planning conversation approach in which the interviewers were trained was based on the POLST conversation model produced by the Coalition for Compassionate Care of California.^17^ The model consisted of the possibility of 3 standardized clinical events occurring in the patient’s current functional state: cardiac arrest during an acute myocardial infarction, severe pneumonia with respiratory failure, and coma after a major stroke. Interviewers were trained to ask patients to confirm their understanding of the information and situations that were presented during the advance care planning conversation. Specifically, interviewers required patients to relay back their interpretation of the information and to explain the reasoning behind their care preferences and their understanding of the likely consequences of the implementation of their preferences.

Each interviewer participated in as many training sessions needed to be considered confident in conducting such a conversation within the role-play scenarios presented in each session. A specific competency checklist to assess interviewers’ readiness to begin interviewing patients was not used; however, we required that both the trainer (E.I.O.V.) and the interviewer under training agreed on the interviewer’s readiness. The trainer focused specific attention on how interviewers framed situations, chose words, confirmed understanding, and recognized evidence of impaired decision-making capacity. Throughout the training sessions, the trainer pointed out how subtle wording choices could unduly persuade patients in a given direction. The minimum number of training sessions was 4 and the maximum was 10.

Interviewers who performed the second interview did not have access to the content of the first interview and were specifically instructed to avoid any conversation with patients about the first interview. Likewise, participants were instructed not to share with the second interviewer any aspect of the first interview because such behavior would jeopardize the research aims.

We used a POLST form that was recently cross-culturally adapted in Brazil^6^ and was mostly based on the 2014 version of the POLST form from the state of Oregon. The Brazilian POLST form has 3 sections for documenting medical orders. Section A pertains to a situation in which the patient is found unresponsive, pulseless, and not breathing and indicates orders to attempt or not to attempt cardiopulmonary resuscitation (CPR). Section B pertains to the level of medical intervention to be provided if the patient has a pulse and is breathing: comfort measures only, limited treatment, or full treatment. Section C pertains to a situation in which the patient has difficulty with oral feeding and indicates orders to provide or not to provide, and for how long to provide, artificially administered nutrition: long-term nutrition by tube, defined trial period of artificial nutrition by tube, or no artificial nutrition by tube. Examples of POLST forms in the United States can be assessed elsewhere.^18,19^ Note that the POLST form is not a questionnaire for patients to fill in which medical treatments they want. Instead, clinicians complete the POLST form according to 1 or more advance care planning conversations they have had with patients, taking into account the patients’ preferences for care related to their current health condition.

### Statistical Analysis

Patients’ demographic and clinical data were described through frequency tables. We described categorical data as absolute numbers and proportions and continuous data as mean and SD or median and interquartile range (IQR), as appropriate.

Statistical analyses follow the principles of classical test theory.^20^ This theory was chosen for its simplicity and efficiency in terms of the sample size needs.^21^ Moreover, some assumptions of the alternative paradigm, the item response theory, do not apply in the context of POLST, such as the local independence between items. To explore the factors associated with the occurrence of disagreements between interviews, we used Fisher exact test when variables were categorical or Wilcoxon rank sum test when variables were continuous.^22^

We assessed interrater reliability using Cohen κ for section A of the POLST form, in which only 2 possibilities of responses exist (ie, attempt or do not attempt CPR), and weighted κ statistic for sections B and C, in which 3 possibilities of answers were given, ranging from less invasive to more invasive treatment options.^23^ We adopted linear weighting as the method for the weighted κ statistic as defined a priori in the study protocol because that strategy was associated with greater simplicity of interpretation.^24^ Nevertheless, following Ben-David’s^25^ recommendation, we performed a sensitivity analysis through the adoption of quadratic weighting to evaluate the robustness of the analyses.

We adopted α = .05 to indicate statistical significance. We used the R software, version 3.3.3 (R Foundation for Statistical Computing)^26^ for all statistical analyses.

We calculated a minimum sample size of 62 participants using the methodology proposed by Rotondi and Donner.^27^ For this calculation, we considered the following guidelines: (1) distribution of marginal proportions in section B of the POLST form of 40% patient preference for comfort measures only, 49% for limited treatment, and 11% for full treatment (we derived these proportions from Hickman et al^28^); (2) values of 0.75 as the lower limit and 0.99 as the upper limit of the 95% CI for the κ statistic; (3) estimated κ value of 90%; (4) presence of 2 interviewers; and (5) α = .05 for statistical significance.

## Results

We included 64 participants in the study, 53 (83%) of whom were patients and 11 (17%) of whom were surrogates. Thirty-five patients (55%) and 8 surrogates (73%) were women. Ten surrogates were children of patients, and only 1 surrogate was a patient’s wife. The Figure shows the flow diagram of participants. The mean (SD) interval of time between interviews was 2 (1.9) days.

**Figure.**
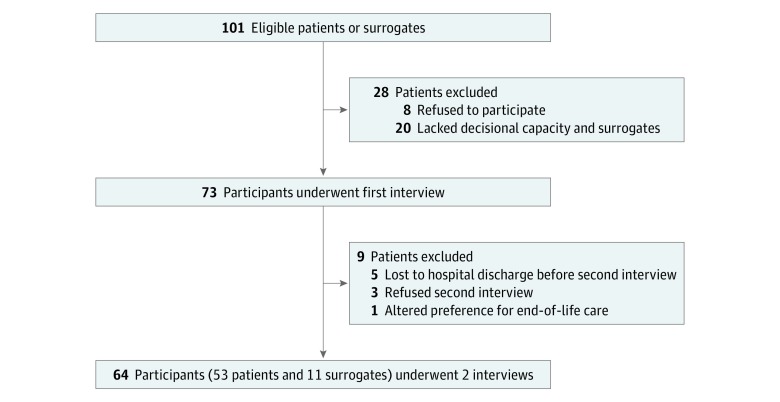
Flow Diagram of the Study Participants

The mean (SD) age of patients was 64 (14) years, and most patients (46 [72%]) self-identified as white and Catholic (40 [62%]). The median (IQR) Charlson comorbidity index was 3 (2-4), and the median (IQR) value for the Palliative Performance Scale was 80% (60%-90%). Further data on the clinical and sociodemographic profiles of patients are shown in Table 1.

**Table 1.** Clinical and Sociodemographic Profile of Patients

Variable	No. (%)
Sex	
Male	29 (45)
Female	35 (55)
Age, mean (SD), y	64 (14)
Race/ethnicity	
Asian	2 (3)
White	46 (72)
Black	16 (25)
Religion	
Buddhism	2 (3)
Catholicism	40 (62)
Spiritism	2 (3)
Evangelicalism	14 (23)
None reported	6 (9)
Years of formal education completed, median (IQR)	4.5 (4-10.5)
Illiterate	2 (3)
Functionally illiterate^a^	2 (3)
Interview performed with	
Patient	53 (83)
Surrogate	11 (17)
Main diagnosis	
Cancer^b^	38 (59)
Cardiovascular disease^c^	12 (19)
Neurodegenerative disease^d^	4 (6)
Other disorder^e^	10 (16)
No. of diagnoses, median (IQR)	3 (2.8-6)
Charlson comorbidity index^f^	
0	1 (2)
1	25 (39)
2	21 (33)
3	17 (27)
Palliative Performance Scale, median (IQR), %^g^	80 (60-90)

Abbreviation: IQR, interquartile range.

^a^ Functional illiteracy was defined as ability to sign name but inability to read a journal or magazine article by self-report.

^b^ Cancer included gastrointestinal, gynecological, breast, pulmonary, urological, and head and neck malignant neoplasms.

^c^ Cardiovascular disease included chronic heart failure, coronary artery disease, and peripheral arterial disease.

^d^ Neurodegenerative disease included Parkinson disease and dementia, such as Alzheimer disease and vascular dementia.

^e^ Other disorder included frailty, hip fractures, liver failure, and other gastrointestinal disorders.

^f^ Higher index means greater burden of comorbidities.

^g^ Palliative Performance Scale score range: 0%-100%, with 0% indicating death and 100% indicating total autonomy plus lack of active illness.

Overall, differences were found in the recorded orders concerning at least 1 section of the POLST form for 5 (8%) of the 64 patients. For 1 participant, the CPR orders changed from the first interview to the second interview. Another participant had discordant orders for CPR and artificial nutrition between the 2 interviews. For 2 participants, the orders for medical interventions and nutrition were different, and for another participant the orders for nutrition changed between the 2 interviews. Table 2 and eTable 1 in the Supplement provide further details regarding the cases of agreement and disagreement between the 2 interviews according to each section of the POLST form.

**Table 2.** Summary of Medical Orders Documented in the POLST and Cases of Disagreement Between 2 Interviews

POLST Form Section	No. (%)		
	First Interview	Second Interview	Disagreement
Section A^a^			2 (3)
Cardiopulmonary resuscitation	48 (75)^b^	48 (75)^b^	
Allow natural death	16 (25)^b^	16 (25)^b^	
Section B^c^			2 (3)^b^
Comfort measures only	0	1 (2)	
Limited treatment	10 (16)	10 (16)	
Full treatment	54 (84)	53 (83)	
Section C^d^			4 (6)
Long-term nutrition by tube	33 (52)	30 (47)	
Defined trial period of artificial nutrition	23 (37)	25 (31)	
No artificial nutrition by tube	8 (12)	9 (14)	

Abbreviation: POLST, Physician Orders for Life-Sustaining Treatment.

^a^ Concerns situations in which patients are found unresponsive, pulseless, and not breathing, and the decisions involve performing cardiopulmonary resuscitation or allowing natural death.

^b^ The proportions and absolute numbers are equal despite the presence of 2 cases of disagreement in medical orders between the 2 interviews because the same number of individuals had their preferences of care recorded in the opposite direction between interviews.

^c^ Concerns any situation in which patients have a pulse and are breathing, and the decisions involve providing medical interventions in general.

^d^ Concerns situations in which patients have difficulty with oral feeding, and the decisions involve providing enteral nutrition or not.

The κ statistics assessing the interrater reliability for each section of the POLST form were high and are presented with their 95% CIs in Table 3. The κ statistic for CPR was 0.92 (95% CI, 0.80-1.00), for level of medical intervention was 0.89 (95% CI, 0.76-1.00), and for artificially administered nutrition was 0.92 (95% CI, 0.83-1.00).

**Table 3.** Raw Interrater Agreement and κ Statistics for Each Section of the POLST Form Between the 2 Interviews of 64 Patients

POLST Form Section	Raw Interrater Agreement, %	κ Value (95% CI)	*P* Value
Section A: cardiopulmonary resuscitation	96.9	0.92 (0.80-1.00)	<.001
Section B: medical intervention	96.9	0.89 (0.76-1.00)	<.001
Section C: artificially administered nutrition	93.8	0.92 (0.83-1.00)	<.001

Abbreviation: POLST, Physician Orders for Life-Sustaining Treatment.

We performed an exploratory comparison of baseline patient characteristics between cases in which at least 1 disagreement in orders between interviews was observed and cases in which complete agreement was found between interviews (eTable 2 in the Supplement). The only statistically significant association we found between patients in those groups involved religion, in which Catholic and Evangelical patients had a smaller proportion of disagreement between interviews compared with patients who were Buddhist, were Spiritist, or reported no religion.

The sensitivity analysis of the weighted κ statistic that used quadratic weighting for sections B and C revealed a κ value of 0.90 (95% CI, 0.79-1.00) for section B and 0.94 (95% CI, 0.88-1.00) for section C.

## Discussion

To our knowledge, this study is the first to assess the interrater reliability of the POLST form completion process after a standardized advance care planning conversation anywhere in the world. The results point toward a high interrater reliability of the POLST paradigm to translate patients’ preferences of care at the end of life into a set of medical orders for life-sustaining treatments. These results are important because they provide evidence supporting the POLST paradigm, which has spread across the United States and has raised interest internationally as a means to promote advance care planning and respect for patients’ values at the end of life.^6^

However, despite the high interrater reliability we found for each section of the POLST form, we found 5 cases of disagreement between the outcomes of the 2 interviews. With regard to the discordance observed in sections B and C of the POLST form, for which there were 3 treatment options representing different degrees of life-sustaining interventions, none of the discordances involved comfort measures in one interview and full treatment in the other. Still, it is certainly disconcerting to find any cases of disagreement for medical orders for life-sustaining treatments within a very short time period. Those disagreements occurred despite our attempts to exclude patients who did not have decision-making capacity or who reported having experienced major clinical or personal events between interviews that changed their perspectives on end-of-life care. Unfortunately, the available data do not make it possible to ascertain with confidence the reasons for those disagreements. The exploratory finding that Catholic and Evangelical patients had less frequent disagreements between interviews than patients who were Buddhist, were Spiritist, or reported no religion must be regarded with great caution because of the low numbers of individuals in the latter categories and the possibility of false-positive associations when conducting multiple statistical tests.^29^ The religious views of patients have been shown to affect their treatment preferences at the end of life, but we could not find studies of the association between religious beliefs and the interrater reliability of instruments used to assess those preferences in a recent systematic review about religious beliefs and major end-of-life issues.^30^

A few hypotheses may explain the cases of disagreement. First, determining decision-making capacity can require some subjective interpretation of its elements, and some patients might not have fulfilled each criterion of that capability during both interviews. A review of 15 instruments used to assess treatment-related decision-making capacity found that the interrater reliability of these instruments was imperfect, with κ values ranging from 0.44 to 0.83.^31^ Second, participants might not have had a consistent opinion about care preferences and changed their minds between the interviews. We believe that the study interviews might have been the first time some participants were asked to consider issues concerning life-sustaining treatments. Third, the interviewers had not been involved with the care of the patients, which could have compromised their ability to identify some subtle inconsistencies in patients’ preferences. Fourth, even though the interviewers were trained in a standardized advance care planning conversation, subtle differences in the way they communicated with participants could have affected the way participants responded to the questions. Fifth, although we generally believe that humans make decisions by rationally weighing risks and rewards, we also believe that reasoning, thoughts, feelings, and decisions are affected by myriad subtle environmental and internal factors of which we are mostly unaware and that sometimes even render us unable to recognize that we have changed our minds.^32,33,34^

Hence, the 5 cases of disagreement in the POLST forms between the 2 interviews emphasize the importance of accurate assessment of decision-making capacity and indirectly support the concept that, ideally, advance care planning conversations should take place within an established patient-clinician relationship in which mutual trust and respect stem from previous experiences.^35^ Ultimately, in real-life situations, such longitudinal relationships between patients, their surrogates, and clinicians are the most important warranty that the content of POLST forms accurately reflects patients’ values and preferences of care. In addition, because miscommunication can occur even between clinicians and patients who have established relationships, the orders on a POLST form must be reviewed frequently to make sure they reflect the current preferences of the patient. In real life, patients receive a copy of their POLST form, representing another assurance that their values are reflected in those medical orders by allowing patients the opportunity to contemplate whether those orders are consistent with their current values. That patients or their surrogates can void POLST forms at any given moment represents yet another aspect of the POLST paradigm that may decrease the possibility of harm from medical orders that become inconsistent with patients’ current preferences of care.

Few studies have assessed the interrater reliability of instruments that document advance care planning conversations. A Malaysian study evaluated the intrarater test-retest reliability of a locally developed questionnaire for assessing individuals’ attitudes and awareness about advance care planning but not patients’ preferences of care at the end of life.^36^ The κ statistic for the items of that questionnaire ranged from 0.74 to 0.95. An Australian study evaluated the interrater reliability of an advance care planning template documenting the care preferences and advance care plans of older adults in residential care facilities.^37^ In the Australian study, 2 independent researchers interviewed 30 older adults within an unspecified period. The κ statistics ranged from 0.73 to 0.79, but no information was provided on the substance of disagreements in specific items of the advance care planning template that was used.

The study has some relevant implications for policy and practice. Although the high interrater reliability that we found offers support for the POLST paradigm, it was not 100% or perfect, highlighting the need to confirm the medical orders on a POLST form on subsequent patient interactions to make sure the orders accurately reflect the patients’ current wishes. Future studies should assess other populations, conduct interviews with somewhat longer intervals of time, and compare preferences of care documented through POLST with different advance care planning strategies. Interrater reliability studies of other advance care planning documents in use are also much needed.

### Limitations

This study has a number of limitations. First, the interval of time between the first and second interviews was short, which may have been associated with some degree of recall bias. Nevertheless, the optimum time interval between interviews for the assessment of interrater reliability depends on the population under study and the construct being measured. The ideal interval of time should not be so long that the construct under study might change but not so short that a recall bias is incurred. Because the population under study was composed of hospitalized patients with serious illnesses, long intervals of time would have been associated with the risk of patients undergoing clinical changes that could affect the construct being measured or of losing the patient from the study because of hospital discharge. Second, we studied a population of inpatients in a university hospital in a middle-income country, which does not reflect other contexts in which POLST has been used. Most studies of POLST were conducted in long-term care facilities in the United States.^3^ On the other hand, although the results may not be generalizable to other populations, they do contribute to the expansion of knowledge about POLST in previously understudied populations. Third, the interviewers were not assessed with a competency checklist to ensure they were adequately prepared to conduct advance care planning conversations. Fourth, despite the high level of interrater reliability that we identified, we cannot completely rule out the possibility that the content of POLST forms was not consistent with patients’ actual values and preferences of care. An assessment of decision quality or the factors in specific treatment preferences was beyond the scope of this study.

## Conclusions

This study appears to provide evidence of high interrater reliability of the POLST completion process, thereby offering further support for this innovative advance care planning paradigm. In addition, the finding that this interrater reliability was not 100% underscores the need to ensure that patients or their surrogates have the decision-making capacity to participate in advance care planning and that a process is in place to confirm that the recorded POLST orders accurately reflect patients’ current treatment preferences.

## Notes

Supplement. **eTable 1.** Comparison of Medical Orders Documented in Physician Orders for Life-Sustaining Treatment (POLST) Forms From the 2 Interviews in Absolute Numbers per Individual Participants (N = 64) **eTable 2.** A Comparison of Cases When There Was at Least One Disagreement Between Interviews and Cases When There Was Complete Agreement Between Interviews (N = 64)
