# Supplementary material for: Evaluation of the Interrater Reliability of End-of-Life Medical Orders in the Physician Orders for Life-Sustaining Treatment Form
Source: JAMA Netw Open. 2019 Apr 12;2(4):e192036. doi: 10.1001/jamanetworkopen.2019.2036 (PMC6481595; doi:10.1001/jamanetworkopen.2019.2036)
Supplement: Supplement. — eTable 1. Comparison of Medical Orders Documented in Physician Orders for Life-Sustaining Treatment (POLST) Forms From the 2 Interviews in Absolute Numbers per Individual Participants (N = 64) eTable 2. A Comparison of Cases When There Was at Least One Disagreement Between Interviews and Cases When There Was Complete Agreement Between Interviews (N = 64) [file jamanetwopen-2-e192036-s001.pdf]

Supplementary Online Content

Lovadini GB, Fukushima FB, Schoueri JFL, et al. Evaluation of the interrater reliability of end-of-life medical orders in the Physician Orders for Life-Sustaining Treatment form. *JAMA Netw Open.* 2019;2(4):e192036. doi:10.1001/jamanetworkopen.2019.2036

eTable 1. Comparison of Medical Orders Documented in Physician Orders for Life-Sustaining Treatment (POLST) Forms From the 2 Interviews in Absolute Numbers per Individual Participants (N = 64)

eTable 2. A Comparison of Cases When There Was at Least One Disagreement Between Interviews and Cases When There Was Complete Agreement Between Interviews (N = 64)

This supplementary material has been provided by the authors to give readers additional information about their work.

©2019 Lovadini GB et al. *JAMA Network Open.*

eTable 1. Comparison of Medical Orders Documented in Physician Orders for Life-Sustaining Treatment (POLST) Forms From the 2 Interviews in Absolute Numbers per Individual Participants (N = 64)

		**Section A: 2nd Interview**	
		Attempt CPR	Do not attempt CPR
**Section A: 1st Interview**	Attempt CPR	47	1
	Do not attempt CPR	1	15

		**Section B: 2nd Interview**		
		Comfort care only	Limited treatment	Full treatment
**Section B: 1st Interview**	Comfort care only	0	0	0
	Limited treatment	1	9	0
	Full treatment for limited period	0	1	53

		**Section C: 2nd Interview**		
		Long-term artificial nutrition by tube	Defined trial period of artificial nutrition by tube	No artificial nutrition by tube
**Section C: 1st Interview**	Long-term artificial nutrition by tube	30	3	0
	Defined trial period of artificial nutrition by tube	0	22	1
	No artificial nutrition by tube	0	0	8

CPR: Cardiopulmonary resuscitation

©2019 Lovadini GB et al. *JAMA Network Open.*

eTable 2. A Comparison of Cases When There Was at Least One Disagreement Between Interviews and Cases When There Was Complete Agreement Between Interviews (N = 64)

**Patients' Characteristics**	**Any disagreement between interviews**	**Complete agreement between interviews**	***P***
Sex, N (%)			0.17[a]
Female	1 (2.9%)	34 (86.2%)	
Male	4 (97.1%)	25 (13.8%)	
Age, mean (SD)	66.7 (14.6)	63.8 (13.9)	0.80[b]
Ethnicity, N (%)			0.10[a]
White	4 (80.0%)	42 (71.2%)	
Black	0 (0%)	16 (27.1%)	
Asian	1 (20.0%)	1 (1.7%)	
Religion, N (%)			0.004[a]
Buddhism	1 (20.0%)	1 (1.7%)	
Catholicism	1 (20.0%)	39 (66.1%)	
Spiritism	1 (20.0%)	1 (1.7%)	
Evangelical	0 (0%)	14 (23.7%)	
No religion reported	2 (40%)	4 (6.8%)	
Years of formal education, median (IQR)	4 (4 to 8)	5 (4 to 11)	0.70[c]
Literacy, N (%)			1[a]
Literate	5 (100%)	55 (93.2%)	
Functionally illiterate	0 (0%)	2 (3.4%)	
Illiterate	0 (0%)	2 (3.4%)	
Interview performed with, N (%):			1[a]
Patient	4 (80.0%)	49 (83.1%)	
Surrogate	1 (20.0%)	10 (16.9%)	
Charlson Comorbidity Index, N (%)			0.21[a]
0	0 (0%)	1 (1.7%)	
1	2 (40.%)	23 (39.0%)	
2	0 (0%)	21 (35.6%)	
3	3 (20.0%)	14 (23.7%)	
Main diagnosis, N (%)			0.07[a]
Cancer	1 (20.0%)	37 (62.7%)	
Cardiovascular	3 (60.0%)	9 (15.3%)	
Neurological	0 (0%)	4 (6.8%)	
Other	1 (20.0%)	9 (15.3%)	
Number of diagnoses, median (IQR)	6 (4 to 6)	3 (2.5 to 6)	0.27[c]
Palliative Performance Scale, median (IQR)	70 (50 to 100)	80 (60 to 90)	0.94[c]

[a] Fisher's test

[b] T test

[c] Wilcoxon rank sum test

©2019 Lovadini GB et al. *JAMA Network Open.*
